# Supplementary figures and images for: Paraplume: A fast and accurate antibody paratope prediction method provides insights into repertoire-scale binding dynamics
Source: PLoS Comput Biol. 2026 Feb 18;22(2):e1013981. doi: 10.1371/journal.pcbi.1013981 (PMC12935307; doi:10.1371/journal.pcbi.1013981)

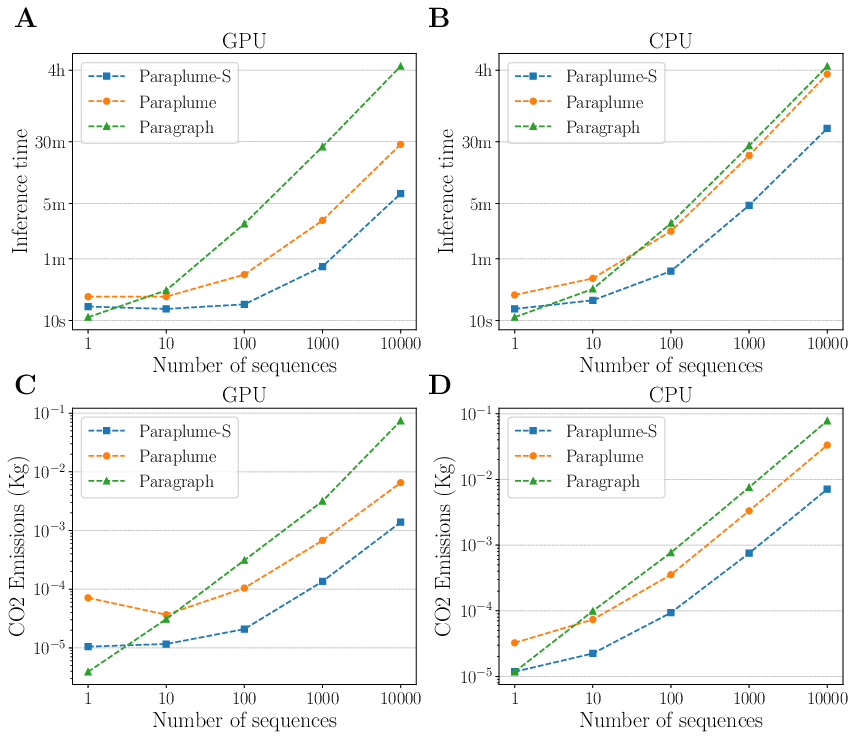

Supplement: S1 Fig — Inference time was compared across different numbers of sequences on an NVIDIA RTX 5000 Ada Generation GPU (A) and 96-core Intel(R) Xeon(R) Gold 6442Y CPUs (B). For Paragraph, 3D structures were generated using AbodyBuilder3, the fastest available structure prediction tool to our knowledge, to ensure a fair comparison. We also compared CO2 emissions using the package codecarbon [43], on GPU (C) and CPU (D). (TIFF) [file pcbi.1013981.s001.tiff]

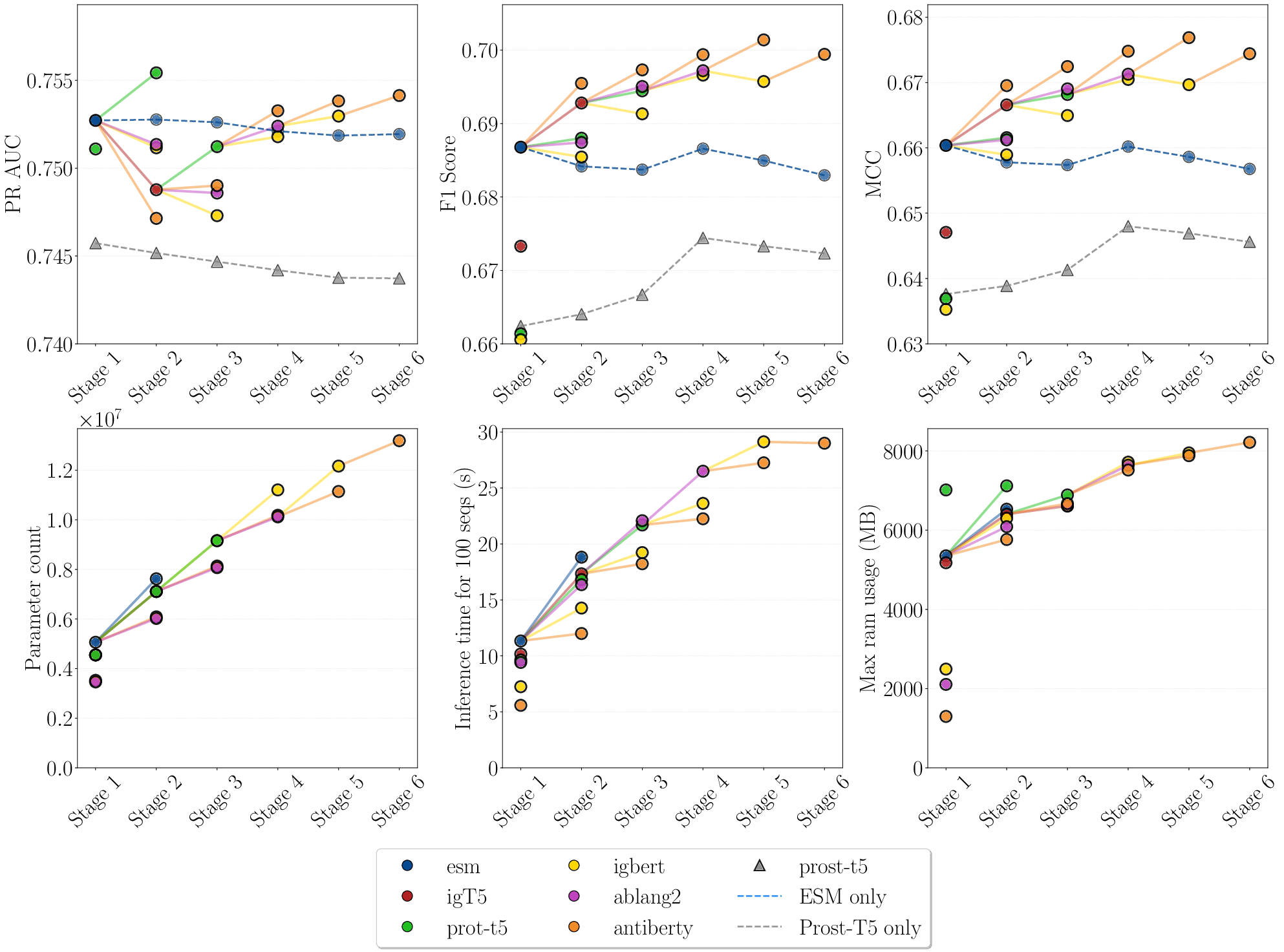

Supplement: S2 Fig — Incremental changes in performance (top row) and computational cost (bottom row) as additional PLMs are sequentially incorporated into the model. All experiments were run on a workstation equipped with two NVIDIA RTX 5000 Ada GPUs (32 GB VRAM), with each run assigned to a single GPU. The PR AUC, F1 score, and Matthews correlation coefficient (MCC) are the evaluation metrics also used in our benchmark with other methods. F1 and MCC are computed using a threshold of 0.5. Results are averaged over 8 seeds and obtained using Paraplume 1.1.0. (TIFF) [file pcbi.1013981.s002.tiff]

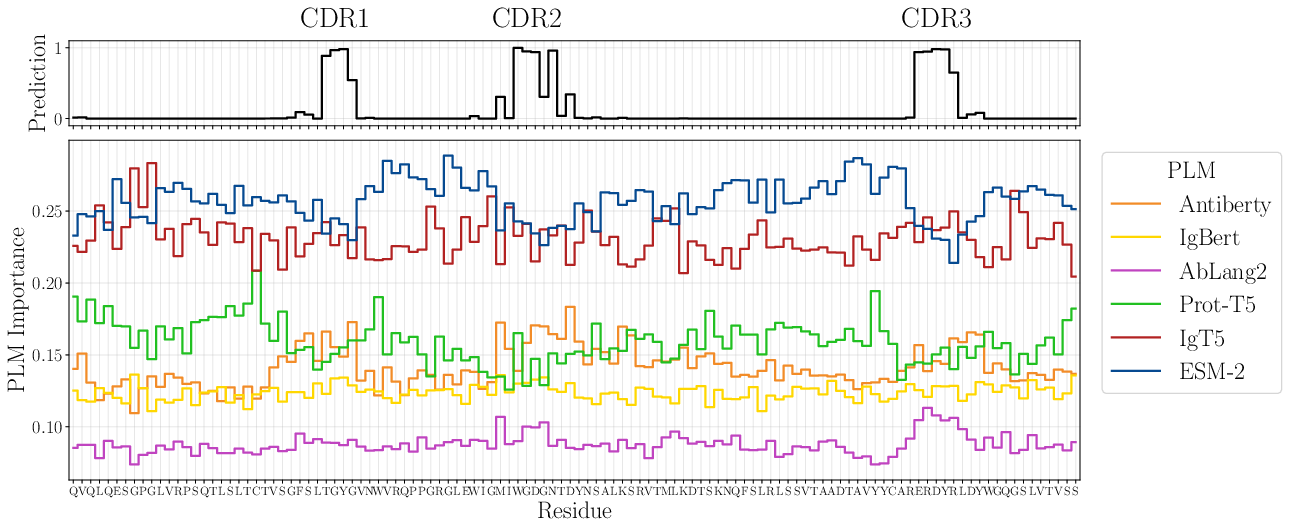

Supplement: S3 Fig — The shown sequence corresponds to the heavy chain of PDB complex 1BVK from the Paragraph test set, with Paraplume trained on the Paragraph training set. (TIFF) [file pcbi.1013981.s003.tiff]

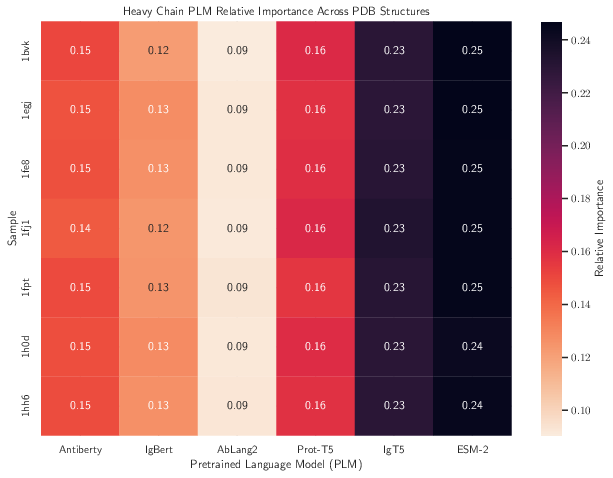

Supplement: S4 Fig — Scores are computed using Shapley values (Shapley-Value–Based Interpretability) and averaged across amino acids within each sequence to obtain a single score per sequence. Complexes shown are from the Paragraph test set with Paraplume trained on the Paragraph training set. (TIFF) [file pcbi.1013981.s004.tiff]

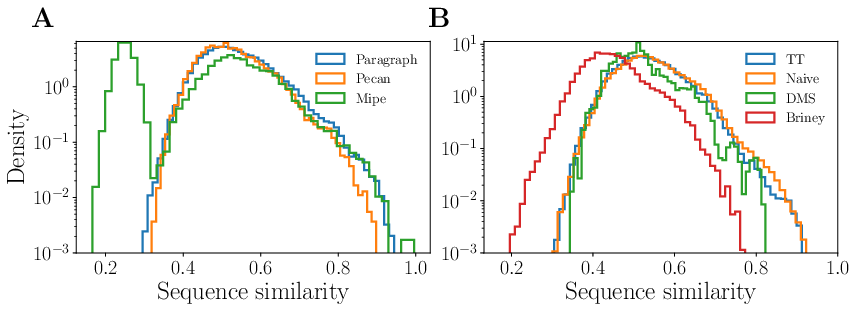

Supplement: S5 Fig — (A) Histogram of sequence similarity between the three benchmark training and test sets. (B) Histogram of sequence similarity between the four analyzed antibody repertoires and the Paraplume training dataset. (TIFF) [file pcbi.1013981.s005.tiff]

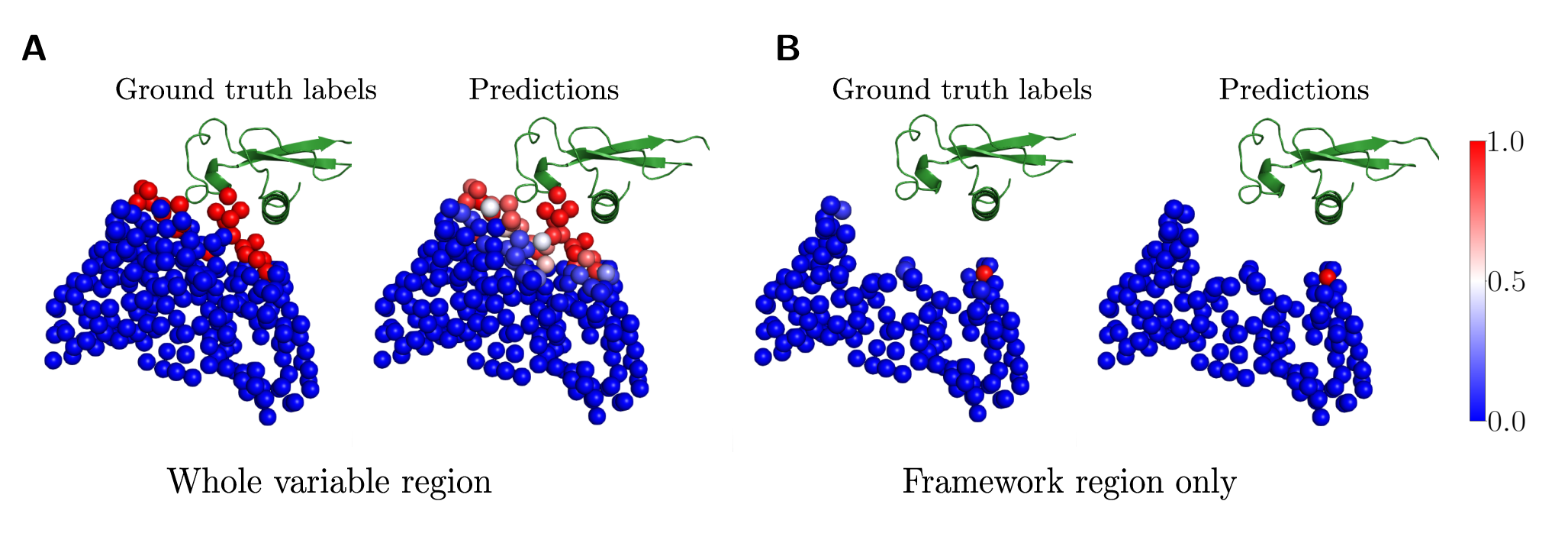

Supplement: S6 Fig — (A) Comparison of ground truth paratope labels (left) and Paraplume model predictions (right) for the full variable region of the 6B0S antibody-antigen complex, which was not included in the training set. For visualization, antibodies were depicted as spheres and the antigen in a cartoon representation (green) in PyMOL [44]. In the ground truth structure, residues forming the paratope are highlighted in red. The colorbar shows the probability of a given amino acid being a paratope residue. For clarity, only the Cα carbon of each residue is depicted. (B) Same structure as in (A) but restricted to amino acids belonging to the framework region. (TIFF) [file pcbi.1013981.s006.tiff]

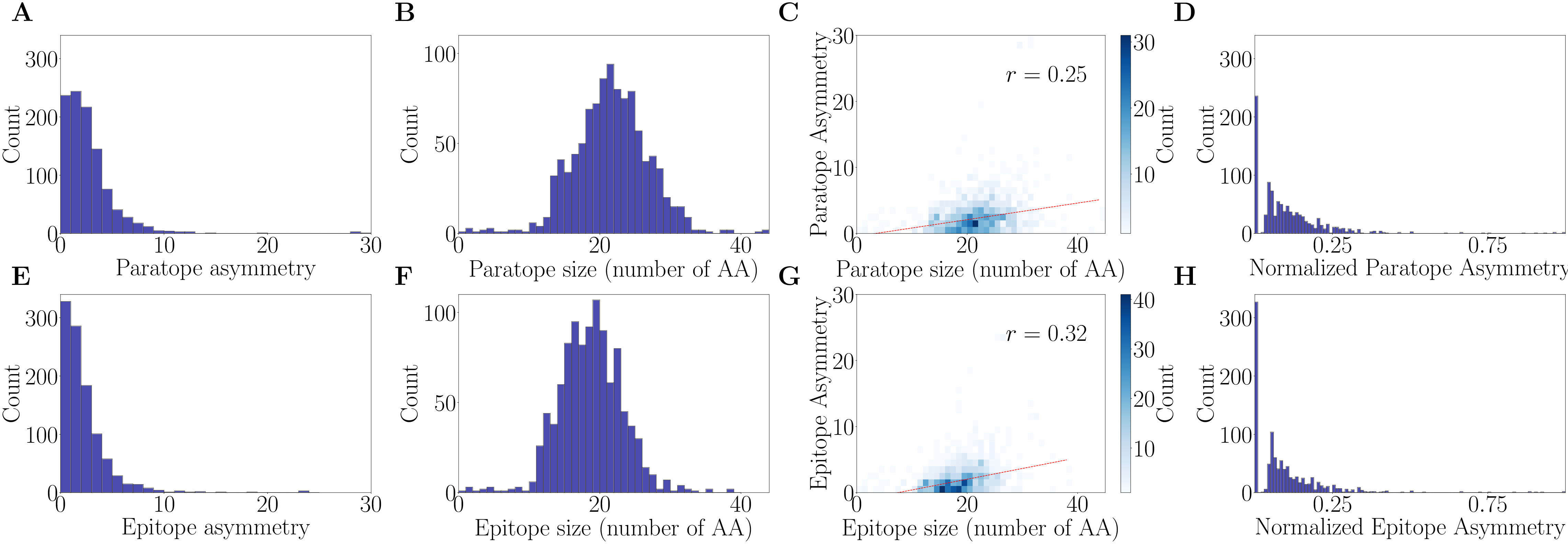

Supplement: S7 Fig — Histograms of the (A) paratope asymmetry and (B) paratope size. (C) Heatmap of paratope asymmetry against paratope size, colored by number of sequences. r is the Pearson correlation coefficient. (D) Histogram of the paratope asymmetry normalized by the paratope size. (E-H) Same as (A-D) but for the epitope. (TIFF) [file pcbi.1013981.s007.tiff]

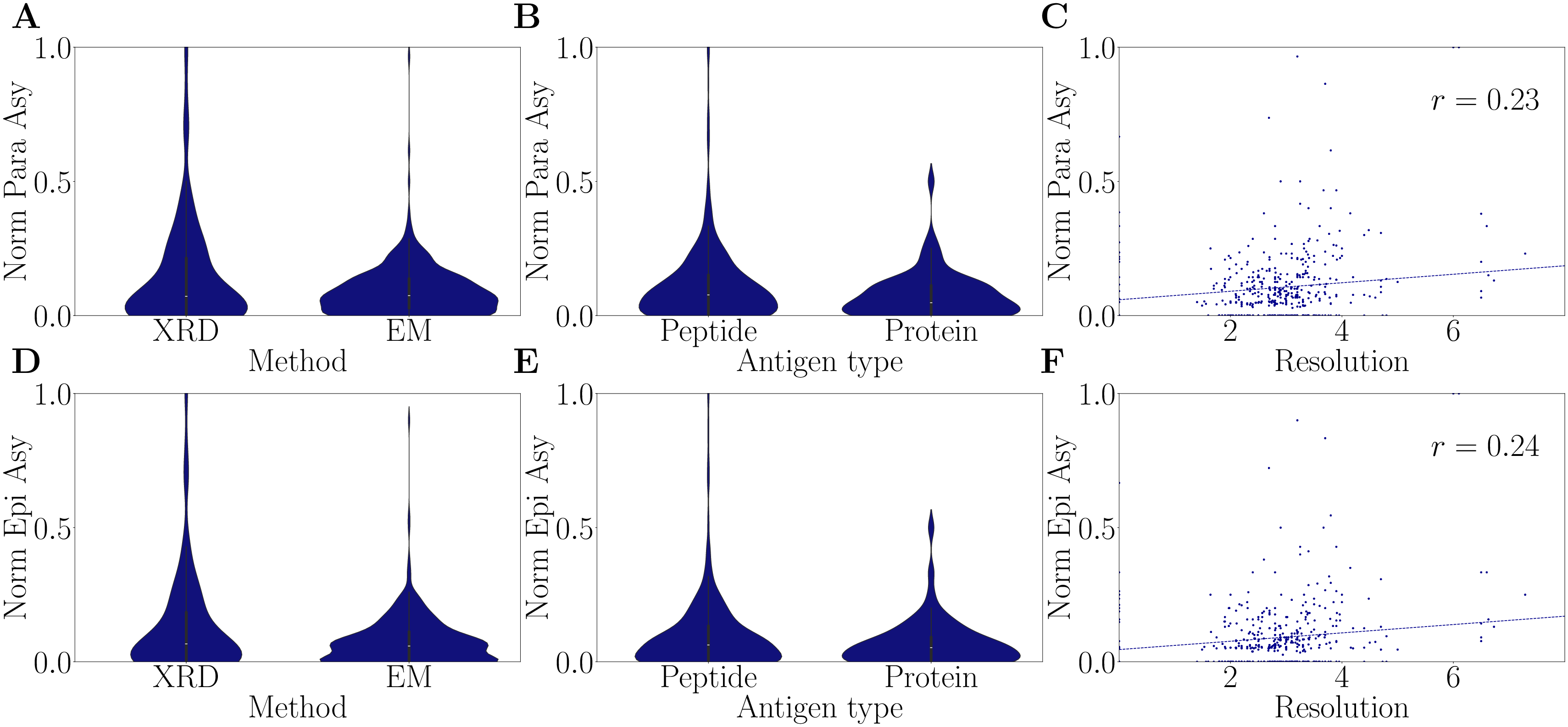

Supplement: S8 Fig — Violin plots of the normalized paratope asymmetry separated by crystallography method (A) and antigen type (B). Normalized paratope asymmetry against PDB resolution (C). (D-F) Same but for the normalized epitope asymmetry. (TIFF) [file pcbi.1013981.s008.tiff]

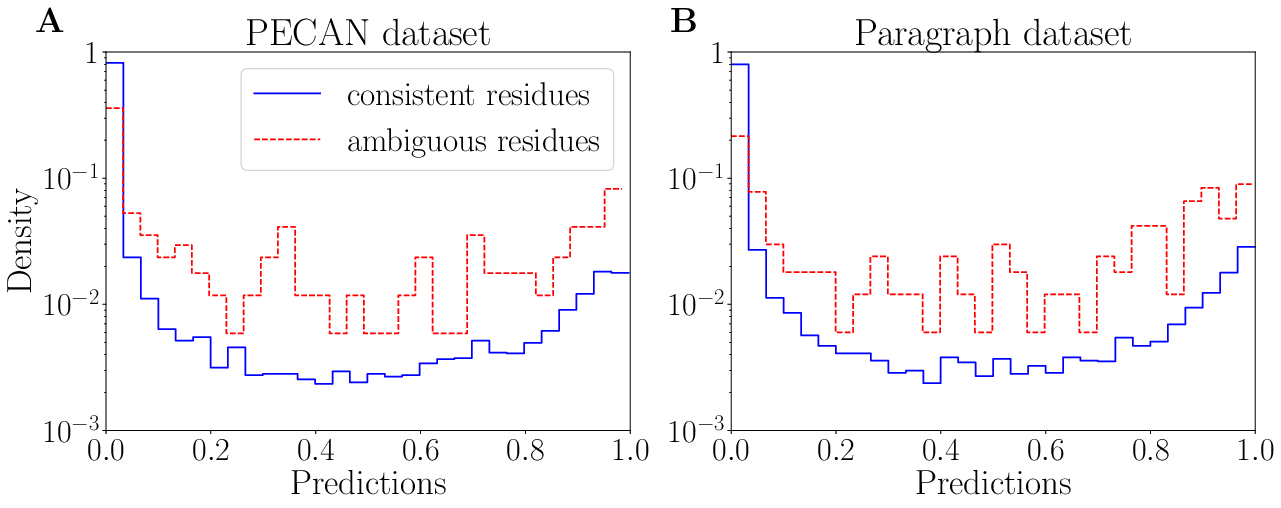

Supplement: S11 Fig — Comparison of Paraplume’s predictions for consistent residues (same paratope label in both arms) and ambiguous residues (different paratope labels in both arms) in the PECAN dataset (A) and Paragraph dataset (B). (TIFF) [file pcbi.1013981.s011.tiff]

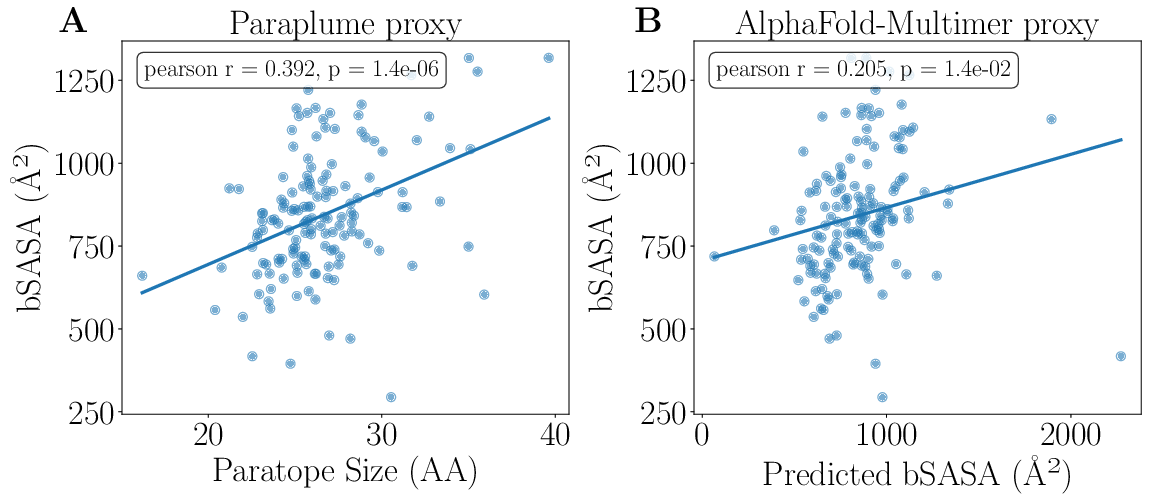

Supplement: S12 Fig — (A) Correlation of paratope size computed using Paraplume with the buried Solvent Accessible Surface Area (bSASA) of ground truth crystal structures of antibody-antigen pairs for all protein antigens of the Paragraph test set, with Paraplume trained on the Paragraph training set. (B) Correlation of the bSASA of the structures predicted using AlphaFold-Multimer and the bSASA of the ground truth crystal structures. Structures were predicted using ColabFold default settings and AlphaFold-Multimer v3. Linear correlation was quantified using Pearson’s correlation coefficient r, and the p-value computed with a two-sided hypothesis test (see pearsonr documentation). (TIFF) [file pcbi.1013981.s012.tiff]

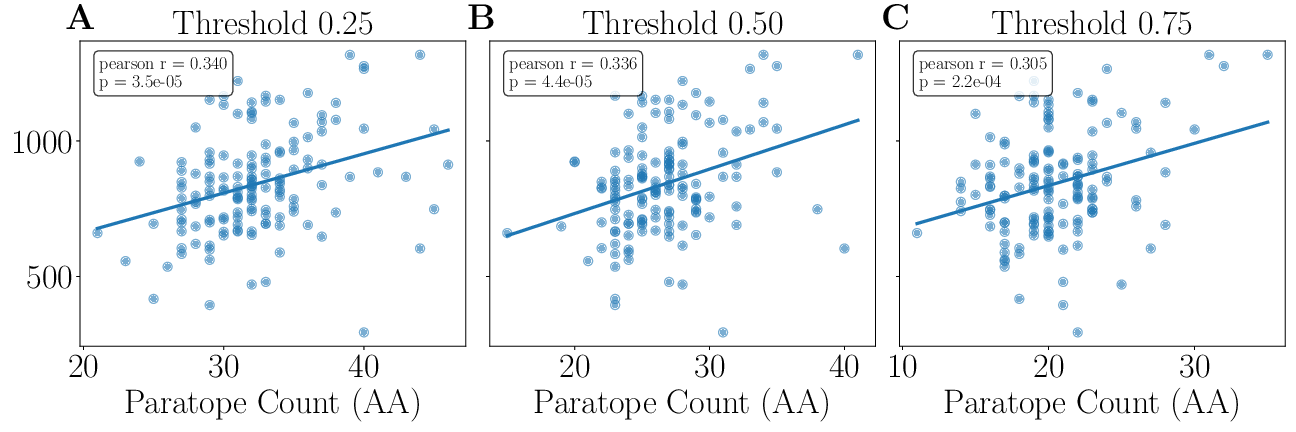

Supplement: S13 Fig — Correlation between thresholded counts of paratope amino acids predicted by Paraplume (probability thresholds: 0.25, 0.5, and 0.75 for A, B, and C panels, respectively) and the buried solvent-accessible surface area (bSASA) from crystal structures of antibody-antigen pairs for all protein antigens of the Paragraph test set, with Paraplume trained on the Paragraph training set. Linear correlation was quantified using Pearson’s correlation coefficient r, and the p-value computed with a two-sided hypothesis test (see pearsonr documentation). (TIFF) [file pcbi.1013981.s013.tiff]

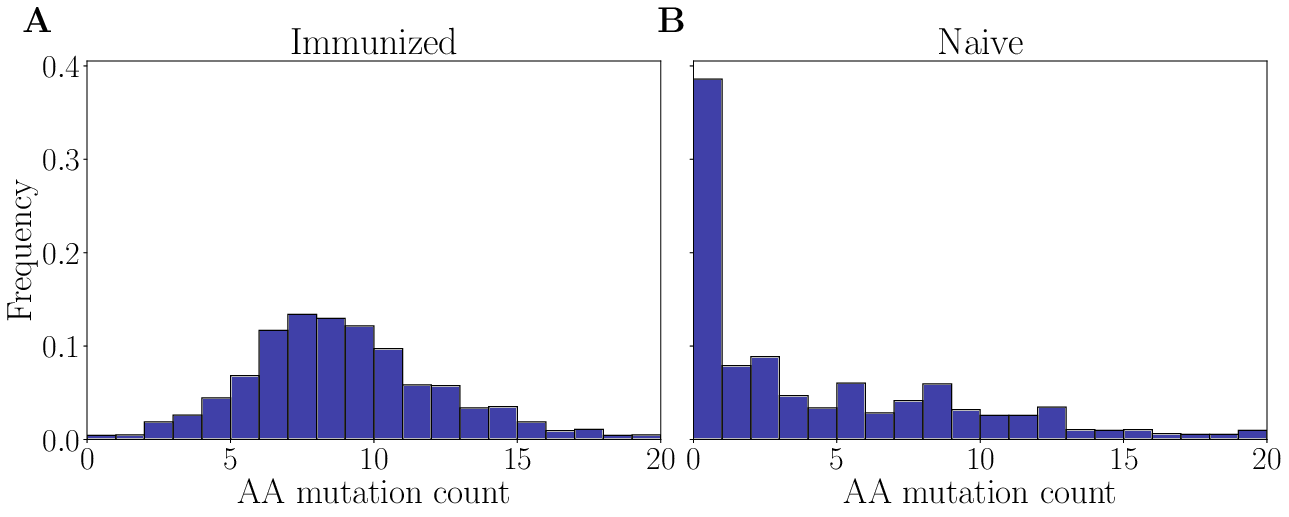

Supplement: S14 Fig — Amino acid mutation count distribution for (A) the immunized mouse antibody repertoire of [30] and (B) the naive mouse antibody repertoire of [31]. (TIFF) [file pcbi.1013981.s014.tiff]

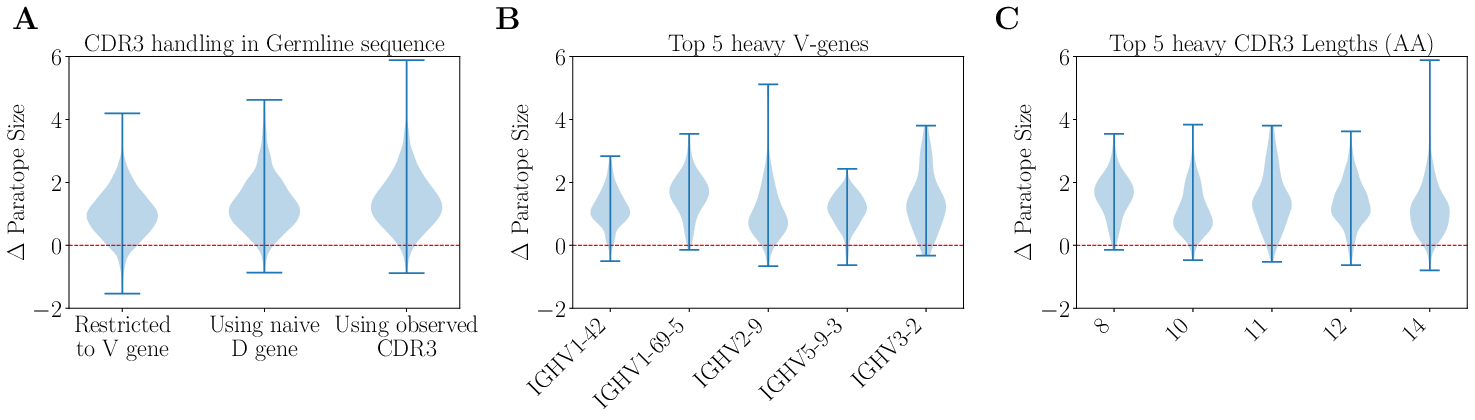

Supplement: S15 Fig — Controls for changes in paratope size between observed IgG sequences and their inferred germlines in mice immunized with tetanus toxoid [30]. Paratope size change is defined as Δ paratope size (observed sequence minus germline). (A) Distribution of Δ paratope size under three CDR3 handling strategies for germline reconstruction: (1) retaining only the V gene (CDR3 and J removed); (2) inclusion of the most likely germline D gene; and (3) use of the observed CDR3 sequence. (B–C) Δ paratope size stratified by the five most frequent V genes (B) and by the five most frequent CDR3 lengths (C). (TIFF) [file pcbi.1013981.s015.tiff]

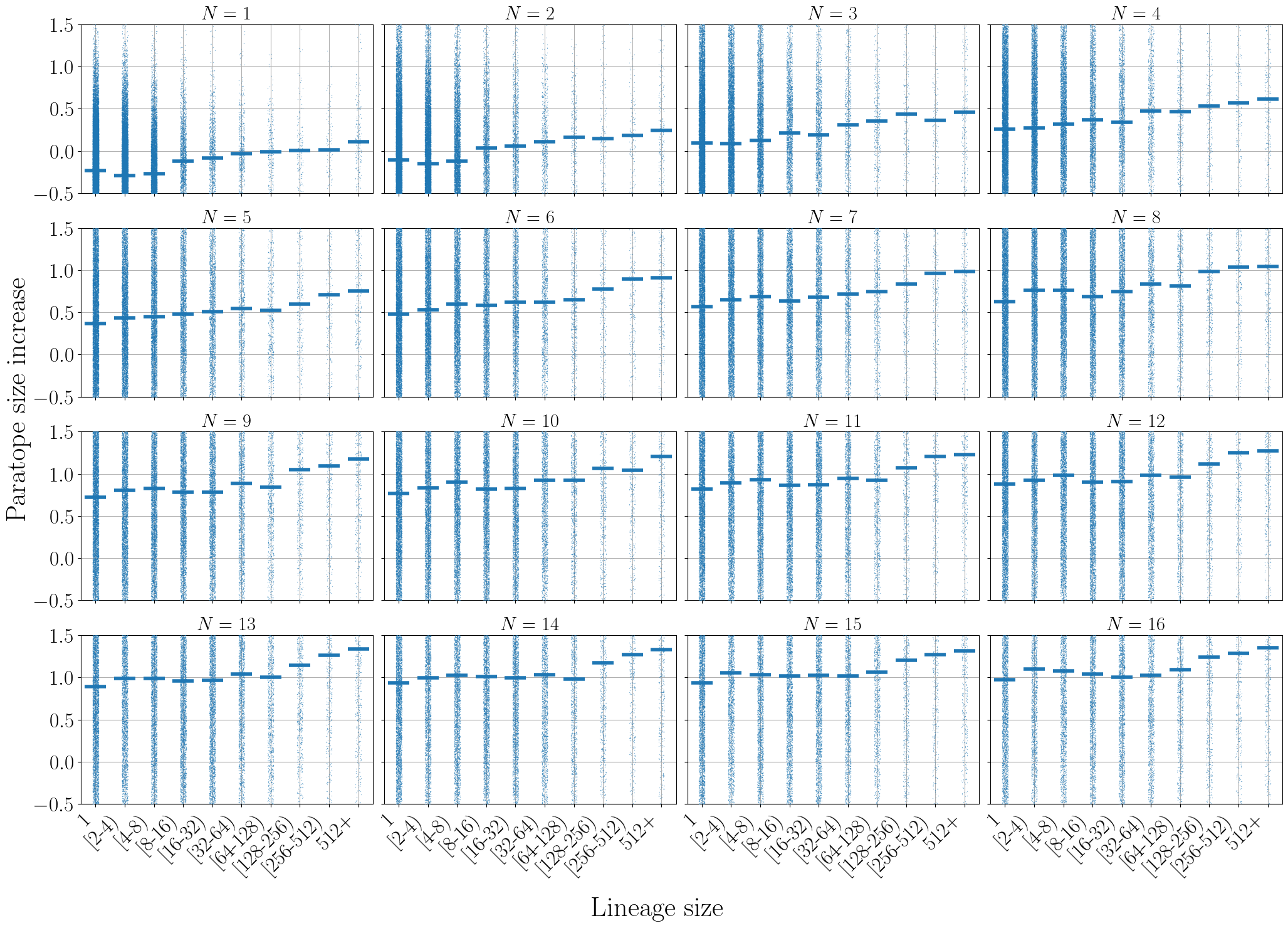

Supplement: S16 Fig — The average in computed over sequences with a fixed number of mutations within the lineage (from N = 1 top left to N = 16 bottom right). Each point is a lineage, and the mean average increase is the thick line. (TIFF) [file pcbi.1013981.s016.tiff]
