# Supplementary material for: Paraplume: A fast and accurate antibody paratope prediction method provides insights into repertoire-scale binding dynamics
Source: PLoS Comput Biol. 2026 Feb 18;22(2):e1013981. doi: 10.1371/journal.pcbi.1013981 (PMC12935307; doi:10.1371/journal.pcbi.1013981)
Supplement: Attachment — Submitted filename: Response_to_Reviewers.pdf [file pcbi.1013981.s023.pdf]

We thank the Editor and the Reviewers for their careful reading of our manuscript and positive assessment. We have answered the Reviewers' comments. Our answers are highlighted in magenta in the "Revised articles with highlighted changes" version of the manuscript.

Reviewer #1: The review is uploaded as an attachment

Reviewer summary of the paper:

- The paper presents an antigen-agnostic method to predict antibody paratopes from sequence alone and a hybrid model that combines sequence and structure-based components by region. It benchmarks against prior tools, analyzes an "upper bound" using antibodies with two arms, and explores biological trends in repertoires. It proposes paratope-weighted pooling of sequence embeddings for downstream tasks such as binder classification and affinity regression. The work is practical and well-motivated. The benchmarking is thoughtful. The repertoire and upper-bound analyses are promising, but several design choices and claims require stricter and more rigorous evaluation.

Major issues:

1. Germline inference uses chimeric sequences that keep the mature CDR3. The "germline" control replaces V and J with inferred germline but seems to retain the mature CDR3. If that's the case, this approach excludes CDR3 mutations from the mutation count and applies paratope prediction to a sequence that never existed in vivo. This can bias the central claims about somatic hypermutation and paratope change. Suggestion: Quantify the share of mutations in CDR3. Repeat key analyses with one or more of the following: mask CDR3 when computing paratope size and weighted pooling, use a consensus naive CDR3 where feasible, or limit to lineages with short CDR3. If none of these are possible, state this limitation prominently in the main text.

We thank the reviewer for raising this important point. As recommended, we repeated our key analyses using several alternative CDR3 controls: (i) masking the CDR3 when computing paratope size (by restricting the analysis to the V genes) and (ii) substituting a consensus naïve D gene. We added a new supplementary figure S15 and a description of the results of this figure to the main text in Section II.F, "Validation on antibody repertoires", page 10, showing that the paratope size increase between the observed sequence and germline sequence is robust to the choice of CDR3 handling strategy.

We fully agree that the treatment of CDR3 in germline inference is inherently challenging. Because CDR3 undergoes extensive somatic hypermutations, its true germline state cannot be reliably reconstructed. For this reason, we chose to preserve the observed (mature) CDR3 in our "germline" controls. This approach ensures that the reconstructed germline sequences remain as close as possible to the experimentally observed receptors in the region where germline inference is most uncertain, and it allows us to focus our mutation counts on positions where we can confidently identify SHMs. Since our analyses compare each observed sequence to its matched germline, we considered it more rigorous to avoid introducing a speculative D that may not reflect the original germline configuration. However it is true that this strategy may exclude CDR3 mutations from SHM quantification and could influence paratope-related analyses. We agree that this is an important

caveat and have now stated it explicitly in the main text: Section II.F, “Validation on antibody repertoires”, page 10.

2. “Identical arms” asymmetry uses a permissive sequence-difference filter. The analysis allows up to high Levenshtein distances between the two arms. Any sequence difference can drive paratope differences and inflate the measured asymmetry that is attributed to biological variability. Suggestion: Recompute the upper bound on the subset with distance equal to zero. Add sensitivity checks with distance less than or equal to two or three if possible. Report asymmetry as a function of arm distance and discuss residual confounding.

We have revised the asymmetry analysis to address this concern by restricting the dataset to arm pairs with a Levenshtein distance of zero. Figure 3A now illustrates the data curation using this strict distance criterion, and Figure 3C has been updated to include only identical arm sequences. In addition, we added a new supplementary Figure S9 to show how arm sequence differences and antigen sequence differences affect the measured asymmetry (Fig. S9A–B). We also performed sensitivity analyses of the paratope asymmetry against the epitope asymmetry in Fig S9 (C-D) (distances ≤2 or ≤3 produced plots nearly identical to distance 0, so we expanded the sensitivity range and now report results for distance bins of 1–10 and 10–20). Note that the upper bound is by definition computed using only amino acids present in both arms. We added a description of the new analysis in the second paragraph of section II.E.

3. The “paratope size” metric is a weak proxy for physical interface size. It sums per-residue paratope probabilities across both chains. This does not reflect physical size on its own, and normalizing by the total size is not sufficient because we need to see what drives the change in the sum or the average. Suggestion: Report thresholded counts at multiple probability cutoffs. Add surface-area weighted measures using predicted structures. And/or, consider heavy-atom counts of contacting residues or counts of hydrogen bond and salt bridge opportunities. Decompose by CDR and framework.

In response to the reviewer’s suggestions, we expanded our analysis to justify our paratope-size metric. First, we added correlation analyses comparing our paratope-size proxy with buried solvent-accessible surface area (bSASA) derived from experimentally solved crystal structures in the Paragraph test set, with Paraplume trained on the Paragraph training set. For comparison, we predicted antibody-antigen complex structures for all test-set sequences using AlphaFold-Multimer v3 with default ColabFold settings. We found that our proxy correlates more strongly with ground-truth bSASA (Pearson r=0.39) than bSASA values computed from AlphaFold-Multimer-predicted structures correlate with their crystal-structure counterparts (Pearson r=0.20), while remaining efficiently computable directly from the model outputs and requiring no antigen sequence information. These results are now presented in Supplementary Figure S12, and discussed in Section II.F (“Mutations increase paratope size”). We also clarify that the paratope size remains only a proxy and better justify its use. Correlations of the ground truth bSASA with thresholded paratope-residue counts at multiple probability cutoffs can now be found in Figure S13. While these thresholded counts provide a more interpretable view of the model’s predictions, the correlation with the ground truth bSASA drops compared to our paratope size proxy, as they inevitably discard some information contained in the full per-residue probability distribution. For this reason, we continue to report the summed paratope

probabilities as our primary metric in the main text, as it effectively captures the integrated signal across thresholds.

4. Claims about specificity from antigen-agnostic predictions are overstated. The manuscript links broader predicted paratopes to higher specificity. Without the antigen, the model identifies where an antibody tends to bind something, not where it binds a given antigen. Suggestion: Pare down the language to association. Add a comparison against an antigen-specific predictor on a matched subset. Report precision and recall trade-offs, average predicted paratope extent, and binder versus non-binder separability.

We thank the reviewer for this important clarification although we were not 100% clear exactly which part of the text was being referred to. We agree that our model, being antigen-agnostic, does not predict specificity toward a particular antigen. Rather, it identifies regions of the antibody that contribute to binding. Our intent is not to claim that larger predicted paratopes imply higher antigen specificity, but that larger predicted paratopes are associated with antibodies drawn from antigen-experienced repertoires compared to naïve repertoires. We revised language in section II.F that could have been interpreted as implying antigen-specific prediction. In particular, the subsection **"II.F. Affinity prediction"** was removed, and the term "predicting epitope specificity" in subsection **"II.F. Antibody classification"** was replaced with "epitope class prediction" to avoid confusion.

5. Paratope-weighted embeddings for affinity regression are not consistently beneficial. The regression results show mixed and often small gains across protein language models and datasets. Some models degrade in some datasets. The current text reads stronger than the evidence. Suggested fix: Report confidence intervals and paired tests for changes in R^2 and rank correlations, especially for Table 4. You can consider using subsampling to create confidence intervals. Keep stronger claims for the classification results where significance is shown and soften the regression claims. Discuss the assumptions, such as disregarding the potential allosteric regulation of binding through residues that are distal to the paratope.

We agree with the reviewer's observation. The affinity-regression results do not show consistent improvements, and the impact of paratope-weighted embeddings varies across datasets and protein language models. Robustly assessing the effect of paratope weighting would require larger and more diverse datasets than the DMS data used here, as well as more extensive statistical analysis. To avoid overstating the benefits of paratope-weighted embeddings for affinity prediction, we have removed this section (previous II.F.affinity prediction) from the manuscript and now focus exclusively on the two classification tasks, where improvements are consistent and statistically supported.

6. Statistical reporting needs to be more explicit in key figures. Some panels, like 4B, show large variance with very small p values and omit test details.

We have updated the caption of Figure 4 (as well as S12 and S13) to explain how the Pearson correlation coefficient (r) and its corresponding p-value were computed.

Minor

1. Why was Paragraph not tested with AF2 as well? Is ABB known to be a higher quality predictor than AF2 for antibodies? If so, please cite papers that show this. Otherwise, please report Paragraph results with AF2 as well.

We thank the reviewer for raising this question. Paragraph was originally evaluated using ABodyBuilder2 (ABB) in the authors' study, which was developed by the same group as an antibody-adapted variant of AlphaFold-Multimer. The performance numbers we cite are taken directly from their benchmarking, which evaluated Paragraph specifically with ABB. As reported in the ABB publication, ABodyBuilder2 represents the state of the art alongside AlphaFold-Multimer, achieving a CDR-H3 RMSD of 2.81 Å, an improvement of 0.09 Å over AlphaFold-Multimer, while being more than one hundred times faster. To ensure full methodological consistency, we reproduced the key analysis on the MIPE dataset following exactly the procedure described in the original Paragraph paper, with ABB3, the most recent iteration from the same group. We now justify this choice in the third paragraph of section II.C.

2. Ensemble size versus practical benefit is not fully justified. The six-PLM ensemble does not dominate on all datasets according to the ablation study and adds computational cost. For example, antiBERTy doesn't seem to help at all based on table S1. Consider having a final model without it (and perhaps also a few others ablated if helpful).

To address the trade-off between ensemble size and practical utility, we performed an additional analysis on the Paragraph benchmark set, the most recent and carefully curated dataset with a 3 Å resolution cutoff and the closest similarity to the final Paraplume training set. We added a new Section II.B to explicitly evaluate PLM complementarity. In the new Supplementary Figure S2, we report both performance gains (PR AUC, F1 score, MCC) and associated computational costs (parameter count, inference time, and memory usage) as PLMs are incrementally added to the ensemble. Specifically, we evaluated all combinations by first testing each of the 6 PLMs individually, then incrementally adding the remaining 5 PLMs one at a time, until all six PLMs were included. Details of this procedure are provided in Section IV.E. We acknowledge that larger ensembles lead to higher computational costs. To address this, we clarify in the third paragraph of Section II.B that Paraplume provides command-line options allowing users to retrain models with any subset of PLMs or on custom datasets. We also emphasize the availability of Paraplume-S, a lightweight alternative to the six-PLM ensemble that uses only ESM embeddings.

3. The hybrid model routing for Paraplume-G needs more clarification. The description of where each component applies by region and how exactly they're fused can be supplemented by a schematic.

We have added clarifications in the second paragraph of section II.D to more explicitly explain how each component is applied to different regions and how their outputs are fused.

4. Show absolute value symbols when plotting magnitude changes.

We have updated Figure 4A to show absolute-value notation when plotting magnitude changes.

5. Fig 4 has the legend overlapping between 4B and 4E.

We have corrected this issue.

6. Putting the codebase on GitHub or an equivalent could make it easier for the reviewers and future users of this work. Zenodo is typically for large datasets associated with the work.

We released both a GitHub repository and a Zenodo archive. The GitHub repository (https://github.com/statbiophys/Paraplume) hosts the software, while the Zenodo record (https://zenodo.org/records/17021232) contains the code needed to reproduce all experiments along with the generated datasets. Both links are provided in the Data Availability subsection of the manuscript.

Overall suggestion and conclusion

- The paper is close to publishable with focused revisions on important claims. I recommend a major revision. The central biological claims should be reanalyzed with corrected germline handling or with strong sensitivity analyses. The asymmetry upper bound should be recomputed on exactly or nearly identical arms or with strict distance controls. The paratope size metric should be replaced or grounded with physical measures. Claims about specificity and regression improvements should be tempered and supported with formal uncertainty. None of these changes requires new experiments. They require careful analysis, clearer reporting, and better phrasing. With these updates, the contribution will be stronger and more reliable.

Reviewer #2: ## Summary

The paper introduces Paraplume, a novel computational method for antibody paratope prediction. Unlike approaches that rely on 3D structures, Paraplume is purely sequence-based. Its architecture leverages embeddings from six different Protein Language Models (PLMs). These embeddings are concatenated and fed into a MLP to predict the paratope probability for each amino acid.

The method was validated on three benchmark datasets (PECAN, Paragraph, MIPE), demonstrating performance that is competitive with or exceeds existing structure-based methods.

Strength

1. **Scalability:** The method's primary strength is its computational efficiency. By avoiding 3D structure modeling, Paraplume is a highly scalable tool practical for analyzing antibody repertoires at a scale of millions of sequences.
2. **Strong Empirical Performance:** Despite its architectural simplicity, Paraplume demonstrates excellent performance, notably outperforming methods that rely on *modeled* 3D structures. This provides strong support for the utility of rich sequence embeddings.

Major Points

While the work has clear merits, several key issues regarding the methodology and experimental validation must be addressed.

1. **Lack of Cost-Benefit Analysis for PLM Embeddings:** The paper claims "complementarity" among the six PLMs but only provides a cost analysis for the final 6-PLM (Paraplume) vs. 1-PLM (Paraplume-S) models. The marginal performance gain of each individual PLM is not weighed against its specific computational cost (e.g., inference time, VRAM usage).

We agree that our original analysis did not sufficiently quantify the trade-offs associated with concatenating PLMs. In response, we added a new Section II.B dedicated to evaluating PLM complementarity and explicitly showing the performance-cost trade-off. For this we implemented an incremental procedure: starting with all six PLMs in Stage 1, we select the best-performing PLM, then sequentially add each remaining PLM in subsequent stages until all six are tested (details in Section IV.E). Using the Paragraph test set, a carefully curated dataset with a 3 Å resolution cutoff that closely matches the dataset used to train the current Paraplume release, Supplementary Figure S2 reports marginal gains in PR AUC, F1, and MCC alongside increases in inference time, VRAM usage, and parameter count. While combining more diverse PLMs does lead to performance improvements, we note in third paragraph of section II.B that several reduced configurations might achieve competitive accuracy at lower computational cost, motivating the release of lighter alternatives and command-line tools for retraining Paraplume on any desired PLM combination.

2. **Confounding Variable: Model Capacity vs. Informational Synergy:** The core methodological claim is confounded. Concatenating six embeddings does not just add "complementary information"; it massively increases the input dimensionality and, consequently, the total parameter count of the MLP (especially in the first hidden layer). The observed performance gain may simply be an artifact of this increased model capacity, rather than true informational synergy.

We thank the reviewer for this important observation. To disentangle the effects of increased model capacity from those of complementarity, we explicitly controlled for input dimensionality by following the reviewer's following suggestion of feeding a single PLM embedding into a wider model. Specifically, to preserve an architecture comparable to that used when concatenating multiple PLMs, we concatenated the same ESM embedding with itself multiple times and compared the resulting performance to that obtained using different combinations of distinct PLMs. As shown by the blue dotted line in Supplementary Figure S2, increasing dimensionality alone leads to performance stagnation, whereas combining embeddings from different PLMs yields consistent gains. This supports the interpretation that the observed improvements arise from complementary information rather than increased model capacity. We clarify this in the first paragraph of section II.B and provide details in section IV.E.

3. **Inadequate Ablation Studies:** Following Point 2, the ablation studies (Table S1) are insufficient. They compare a 6-PLM model to 1-PLM models *with the same MLP architecture*. A proper control is missing: a 1-PLM model (e.g., Paraplume-S) fed into a

"wider" or "deeper" MLP with a parameter count equivalent to the full Paraplume model. Without this control, the central claim of "complementarity" is unsubstantiated.

We agree that increased model capacity could confound the apparent benefits of concatenating multiple PLM embeddings. To address this, we follow the suggestion by including a control in which a single PLM (ESM) is concatenated with itself multiple times. This control shows that the observed gains are not merely due to increased model capacity: stacking multiple copies of the same embedding yields substantially worse performance than combining different PLMs, confirming that the improvements reflect PLM complementarity. We now present this control in Supplementary Figure S2 (blue dotted line), discuss it in the first paragraph of Section II.B, and provide methodological details in Section IV.E.

4. **Omission of Key Model Comparisons:** The paper omits comparisons to a critical third class of models: structure-aware sequence models (e.g., ProstT5, TM-vec). These models explicitly integrate 3D knowledge during pre-training but require only sequence input at inference. To validate the claim that Paraplume's *implicit* structural information is superior, it must be benchmarked against these *explicit* structure-aware SOTA methods.

We thank the reviewer for bringing this important class of models to our attention. In response, we evaluated Paraplume's performance when incorporating the structure-aware ProtST5 models. As part of our ablation study explained section II.B and IV.E, we applied the same control used before to account for increased model capacity by stacking multiple copies of the ProstT5 embedding. We found that stacking ProstT5 embeddings performed substantially worse than all combinations of PLMs tested in our ablation study. We also discuss in the second paragraph of section II.B the potential use of ProtST5 in combination with the existing PLMs. However, we did not include it in Paraplume due to concerns about data contamination: ProtST5 relies on AlphaFold-predicted structures, and AlphaFold was trained on structures that overlap with the test sets used in our benchmark.

5. **Insufficient Case Study:** The case study in Figure 2 demonstrates Paraplume's success but not its *superiority*. A more compelling case would compare Paraplume against a structure-based method (e.g., Paragraph-ABB) on an antibody where the underlying 3D structure model (e.g., ABodyBuilder) is known to fail (e.g., a complex CDR loop), showing that Paraplume succeeds where the structure-based method fails.

We thank the reviewer for the suggestion. The original purpose of Figure 2 was to highlight Paraplume’s ability to make predictions in framework regions, which are not covered by structure-based methods such as Paragraph that focus on the CDRs. However, we agree that this example was not sufficiently informative for a main figure and have therefore moved it to Supplementary Figure S6. Figure 2 has been replaced with a new analysis showing average Paraplume predictions across IMGT positions for 218 unseen sequences. This figure demonstrates that Paraplume captures the DE loop despite its location in the framework region. We also include PLM importance scores computed using Shapley values, showing increased contributions from antibody-specific PLMs in antibody-specific loop regions, which further supports PLM complementarity. The corresponding text is the last paragraph of section II.B.

6. **Lack of Model Interpretability:** The model is treated as a "black box." The claim of "complementarity" is an untested hypothesis. The authors should apply feature attribution methods (e.g., SHAP, Integrated Gradients) to the trained MLP. This would reveal which PLMs the model relies on for different predictions (e.g., CDR3 vs. framework regions) and provide mechanistic evidence for the complementarity claim.

We thank the reviewer for this valuable comment, which helped improve both the manuscript and the method. We agree that using an MLP, while lightweight and scalable, comes at the cost of interpretability. To provide interpretability within this architecture, we applied SHAP analyses to quantify the contribution of each PLM to the model's predictions, providing insight into how different embeddings contribute across sequence regions. We now report PLM importance scores averaged over IMGT positions for 218 unseen sequences in Figure 2. In addition, we added visualization tools that display PLM contributions at the sequence and residue levels, illustrated in Supplementary Figures S3 and S4. These analyses are described in the final paragraph of Section II.B, with methodological details on Shapley values provided in Section IV.F.

7. **Omission of Interpretable Architectures:** The choice of an MLP, while simple, sacrifices model interpretability. Other architectures, such as those using attention mechanisms (e.g., VenusVaccine), provide clear, residue-level explanations for their predictions. The authors should justify why an opaque MLP was chosen over more transparent alternatives and demonstrate some aspect of their model's interpretability.

We acknowledge that the use of an MLP limits interpretability in the last paragraph of Section II.F, and we further discuss in the Discussion that attention-based models provide more direct residue-level interpretability and represent a promising direction for future work. To address interpretability within our current architecture, we follow the reviewer's suggestion and apply Shapley value analyses to quantify the contributions of individual embedding components. These results are presented in Figure 2 and Supplementary Figures S3 and S4, with methodological details provided in Section IV.E.

Minor Points

1. **Incorrect Terminology in Figure:** In Figure 1B, the label "Pretrained LLMs" is used, maybe "Pretrained PLMs"?

We have corrected this typo.

2. **Figure Quality:** Figures 1 and 2 require improvement. Figure 1B is overly simplistic; a more informative schematic might briefly illustrate the different types of PLMs used. Figure 2 is a basic render; a more aesthetic visualization would be more impactful.

We have revised Figure 1B to more clearly illustrate the different types of PLMs used in our model. Figure 2 has been moved to the Supplementary Information and replaced in the main text with a more informative and visually impactful figure.

3. **Accessibility of Citations:** The submitted PDF lacks active hyperlinks for citations.

We added active hyperlinks for citations.

4. **Critical Link Error:** The Zenodo link provided on Page 4 (.../records/17277448) contradicts the link provided on Page 18 (.../records/17021232).

We now have one consistent zenodo link.

Reviewer #3: This manuscript presents Paraplume, a fast and accurate paratope prediction framework that enables repertoire-scale analyses of antibody binding, and it demonstrates solid innovation, good readability, and a rich, convincing set of experiments. The following points require clarification:

1.The Introduction and Discussion could more clearly articulate what is conceptually new relative to existing paratope predictors such as Paragraph, PECAN, MIPE and Parapred. In particular, please spell out which aspects are the true contributions (multi-PLM sequence-only paratope prediction, paratope-weighted embeddings, repertoire-scale analyses, asymmetry-based upper bound) and which are incremental improvements in benchmarking.

We thank the reviewer for this helpful suggestion. We have revised both the Introduction (last paragraph) and Discussion (fourth paragraph) to more explicitly articulate the conceptual contributions of our work relative to existing paratope predictors such as Paragraph, PECAN, MIPE, and Parapred.

2.The manuscript would benefit from a more detailed description of how sequence redundancy and potential train–test leakage are controlled across the PECAN, Paragraph, MIPE, and expanded SAbDab datasets (e.g., sequence-identity thresholds, overlap between ABB3 / AlphaFold training data and the benchmark splits, and between Paraplume's training set and the repertoires analyzed downstream). Given that you already note ABB3 training/test overlap for Paragraph on MIPE, a systematic treatment of these issues is important for a fair comparison.

We thank the reviewer for this very important comment and fully agree that a quantitative assessment of sequence redundancy between training and test sets is essential. For Paraplume, no leakage can occur from ABB3/AlphaFold training data because Paraplume is a sequence-only model and does not rely on any structure-prediction components. Its benchmarks therefore do not involve any structure-prediction training sets.

For Paraplume-G, all benchmarks use experimentally determined crystal structures, so leakage from structure-prediction training sets is not possible.

For Paragraph and PECAN, we rely on the procedures described in the original papers: both use ABodyBuilder and ABlooper while preventing the use of sequence-identical templates, which mitigates template-based leakage. The only scenario where leakage arises as described in the manuscript is when we retrained Paragraph with ABB3 for the MIPE dataset; however, any such leakage would affect only the competitor's method, not Paraplume. Our intent in retraining was to put Paragraph under the strongest possible conditions. We clarified this point in the first paragraph of the Results section II.C, and now provide distributions of sequence similarity between the train and test splits for the PECAN, Paragraph, and MIPE benchmarks in Supplementary Figure S5A. Finally, to ensure that downstream repertoire analyses are not influenced by training data, we verified that no sequences in Paraplume's training set overlap with any repertoire sequences analyzed later in the paper. We show in figure S5B the pairwise distances between the final Paraplume training set and all downstream repertoires.

> 3.While Section IV. A briefly justifies the use of six PLMs and mentions an ablation in Table S1, the main text does not fully convey how much each embedding source contributes versus the added computational cost. I suggest moving at least a subset of these ablation results into the main Results, and clarifying whether a learned linear combination or dimensionality reduction of concatenated embeddings was tested as an alternative to the very high-dimensional concatenation.

We agree that the main text should more clearly communicate each PLM's contribution relative to its computational cost. To address this, we developed a procedure to assess the incremental contribution of additional PLMs, detailed in Section IV.E. The results are now reported in a new Section II.B and Figure S2, which show, for each PLM addition, both performance gains (PR AUC, F1, MCC) and the associated computational costs (VRAM usage, inference time, parameter count). This provides a transparent view of the trade-offs involved in expanding the embedding set. We also explored dimensionality reduction of the concatenated embeddings via PCA; however, this led to a substantial drop in performance, as discussed in the third paragraph of Section II.B, indicating that high-dimensional concatenation is necessary to preserve predictive power.

> 4.The analysis of paratope/epitope asymmetry in antibodies binding identical antigens is interesting, but the resulting ~0.95 F1 "upper bound" is presented somewhat strongly as a performance ceiling. Please clarify that this bound depends on the chosen distance cutoff, labeling protocol, and dataset composition, and consider showing the full distribution of arm-to-arm F1/PR AUC across complexes to emphasize variability rather than a single number.

We have revised the manuscript to clarify that the ~0.95 F1 value is not intended as a universal performance ceiling, as it depends on the labeling protocol and the composition of the dataset (third

paragraph of section II.E). We also now report the full distribution of arm-to-arm F1 and MCC values across the paragraph dataset in figure S10. (Regarding PR AUC: this metric is defined for probabilistic predictions, so it cannot be computed for the binary arm-to-arm labels used in this analysis.)

5.The conclusion that somatic hypermutation leads to larger paratopes in mouse and human repertoires is compelling but may be influenced by confounders such as total variable-region length, CDR3 length, or V-gene usage. Since "paratope size" is defined as a sum over per-residue probabilities, it should be made clearer how you control for length and composition (e.g., via normalized paratope size, length-stratified analyses, or multivariable models including SHM count and CDR length).

The reviewer raises an important point. In our analyses, we always compare the paratope size of each sequence to that of its matched germline. As a result, factors such as variable-region length, CDR3 length, or V-gene usage are identical between the germline and the observed sequence and therefore do not directly influence the observed increase in paratope size. We acknowledge, however, that the background sequence on which mutations occur could in principle affect how paratope size increases. To address this, we report in figure S15 the distribution of paratope size increases for the five most common heavy V genes and the five most common heavy CDR3 lengths, demonstrating that the increase is consistent across diverse sequence backgrounds. Additionally, for the correlation between mutation counts and paratope size, we show the paratope size of the corresponding germlines in blue (Figures 4E and 5B), illustrating that germline paratope size does not increase with the number of mutations carried by the observed sequence. This ensures that our analysis is not biased by some V genes having naturally larger germline paratopes than others. We clarified the manuscript in the first paragraph of section II.V "validation on antibody repertoires" page 10.

6.The Methods state that hyperparameters were selected on the Paragraph dataset and then reused for all benchmarks, and that multiple seeds with early stopping were employed, but these details are somewhat scattered. For reproducibility, please explicitly summarize in one place the training protocol (number of seeds, early-stopping criterion, epochs, optimizer settings, batch sizes, and hardware) and ensure that the GitHub repository mirrors this exact configuration so that readers can reproduce the main tables.

To improve clarity and reproducibility, we have added a dedicated subsection in the Methods that explicitly summarizes the full training protocol, including the number of seeds used, early-stopping criteria, total epochs, optimizer and learning-rate settings, batch sizes, and hardware specifications, which are mirrored in the github repository.

7.The authors propose an effective strategy. In future work, widely developed deep learning techniques and their application to more complex scenarios (DOI:

10.1145/3664647.3681673, DOI: 10.1109/JBHI.2024.3357979) could be further explored.

We thank the reviewer for the suggestion. While the specific papers cited address application areas different from ours, we note in the Discussion that attention-based methods could be explored in future work to potentially improve prediction performance and provide enhanced residue-level interpretability.
