# Supplementary material for: Paraplume: A fast and accurate antibody paratope prediction method provides insights into repertoire-scale binding dynamics
Source: PLoS Comput Biol. 2026 Feb 18;22(2):e1013981. doi: 10.1371/journal.pcbi.1013981 (PMC12935307; doi:10.1371/journal.pcbi.1013981)
Supplement: Attachment — Submitted filename: Response_to_Reviewers_round2.pdf [file pcbi.1013981.s024.pdf]

We thank the Editor and the Reviewers for their careful reading of our manuscript and positive assessment. We have answered the Reviewer's comments. Our answers are highlighted in magenta in the "Revised articles with highlighted changes" version of the manuscript.

Major comments:

1. Regarding the identical arms asymmetry, the sensitivity bins of 1 to 10 and 10 to 20 are very broad. They can mask a near-linear trend in the 1 to 5 range. Also, Fig S10 suggests that a considerable number of arm pairs have perfect MCC and F1. In this case, you should report the upper bound on distance zero cases (maybe perfect MCCs and F1 scores come from there) and separately report MCC and F1 as a function of narrowly binned distances (not 1 to 10 but much smaller increments) between the two arms of the antibody. The sequence differences may be confounding MCC and F1s since you're averaging across all of the sequence distances. This should be a straightforward reanalysis.

We thank the reviewer for this detailed and thoughtful comment. In response, we have extended our analysis to address the concerns regarding binning and potential confounding by arm sequence differences.

First, we now report epitope asymmetry as a function of paratope asymmetry using finer arm-difference bins. Specifically, we include analyses where the arm difference ranges from 1 to 4 (Figure S9E-H), in addition to the broader ranges of 1-10 and 11-20 shown in Figure S9C-D, the zero-difference case presented in the main text (Figure 3C), and the correlation between paratope and epitope asymmetry with arm difference (Figure S9A-B).

Second, to address the reviewer's suggestion regarding an upper-bound analysis, we repeated the evaluation using only antibodies with an arm distance of zero (described on page 10). This resulted in only minor changes to the reported upper bounds: F1 scores and MCCs across the three datasets changed from 0.947, 0.944, 0.953, 0.949, 0.961, and 0.958 to 0.956, 0.953, 0.955, 0.951, 0.955, and 0.952, respectively. This is mainly because the original test datasets were already curated for high quality by the original authors and therefore contained mostly antibodies with identical arms. As a result, the number of antibodies included decreased from 80 to 67 for the Paragraph dataset, from 66 to 51 for the Pecan dataset, and from 18 to 13 for the MIPE dataset.

Additional factors also explain why restricting the analysis to zero arm difference does not substantially change the results: (1) sequence arm difference correlates only weakly with paratope asymmetry (Figure S9A–B); (2) even antibodies with identical arms can exhibit substantial paratope asymmetry (Figure S9A–B and Figure 3); and (3) by construction, the confusion-matrix terms required to compute the F1 score can only be defined over residues present in both arms (i.e., identical amino acids at the same IMGT position). As a result, residues present in only one arm do not contribute to the F1 score, even for antibodies with larger arm differences. However, missing or altered residues in a PDB structure could influence the positions of surrounding residues and therefore their paratope labels. For completeness, we now include histograms of MCC and F1 scores for antibodies in the

Paragraph test set, comparing antibodies with zero arm difference (blue) and strictly positive arm differences (orange) in Figure S10.

Minor comments:

1. Regarding the use of embeddings and the downstream tasks, I think you shouldn't remove the negative result but instead change the language. It is important for the reader to know that your proposed weighted embedding is not a one-size-fits-all method but can yield performance improvements in some tasks. I see scientific value in all the results and in transparently reporting them. You can cite that as a limitation of the method. If you discard the negative results, the reader will mistakenly think that the paratope-weighted embeddings will work for whatever relevant downstream task they have.

We thank the reviewer for this positive feedback. We have moved these results back to the main text and adjusted the wording, taking into account the reviewer's earlier comments from the first round of revision. We clearly state on pages 15-16 that paratope-weighted embeddings can be deleterious in certain contexts and show that the affinity prediction results are not statistically significant. We also added a paragraph to the Discussion on page 17 describing the limitations of this approach, in particular that it can down-weight residues that are distal to the paratope but still contribute to binding.

2. Please specify which ESM-2 model size was used. I don't see this clearly mentioned, but there are a few different ESM-2 options with different sizes and embedding dimensions.

We added these clarifications in the Methods section page 18.

3. When you make scatter plots, it would be helpful not to set alpha (or opacity) to 1 so that the reader can understand the density of the dots in a given cluttered region.

In response, we decreased the opacity of all scatter plots in Figure S9 to improve visualization.

4. In the beginning of page 10/29, you refer to Fig S8C, but it should be Fig S9C based on the supplementary figure tiff files attached.

We thank the reviewer for pointing out this typo. The figure now referenced is Figure 3C, showing the epitope-paratope asymmetry correlation on antibodies with identical arms.
