# Supplementary material for: Paraplume: A fast and accurate antibody paratope prediction method provides insights into repertoire-scale binding dynamics
Source: PLoS Comput Biol. 2026 Feb 18;22(2):e1013981. doi: 10.1371/journal.pcbi.1013981 (PMC12935307; doi:10.1371/journal.pcbi.1013981)
Supplement: Attachment — Submitted filename: peer review for Paraplume.docx [file pcbi.1013981.s022.docx]

Reviewer summary of the paper:

- The paper presents an antigen-agnostic method to predict antibody paratopes from sequence alone and a hybrid model that combines sequence and structure-based components by region. It benchmarks against prior tools, analyzes an “upper bound” using antibodies with two arms, and explores biological trends in repertoires. It proposes paratope-weighted pooling of sequence embeddings for downstream tasks such as binder classification and affinity regression. The work is practical and well-motivated. The benchmarking is thoughtful. The repertoire and upper-bound analyses are promising, but several design choices and claims require stricter and more rigorous evaluation.

Major issues:

1. Germline inference uses chimeric sequences that keep the mature CDR3. The “germline” control replaces V and J with inferred germline but seems to retain the mature CDR3. If that’s the case, this approach excludes CDR3 mutations from the mutation count and applies paratope prediction to a sequence that never existed in vivo. This can bias the central claims about somatic hypermutation and paratope change. Suggestion: Quantify the share of mutations in CDR3. Repeat key analyses with one or more of the following: mask CDR3 when computing paratope size and weighted pooling, use a consensus naive CDR3 where feasible, or limit to lineages with short CDR3. If none of these are possible, state this limitation prominently in the main text.
2. “Identical arms” asymmetry uses a permissive sequence-difference filter. The analysis allows up to high Levenshtein distances between the two arms. Any sequence difference can drive paratope differences and inflate the measured asymmetry that is attributed to biological variability. Suggestion: Recompute the upper bound on the subset with distance equal to zero. Add sensitivity checks with distance less than or equal to two or three if possible. Report asymmetry as a function of arm distance and discuss residual confounding.
3. The “paratope size” metric is a weak proxy for physical interface size. It sums per-residue paratope probabilities across both chains. This does not reflect physical size on its own, and normalizing by the total size is not sufficient because we need to see what drives the change in the sum or the average. Suggestion: Report thresholded counts at multiple probability cutoffs. Add surface-area weighted measures using predicted structures. And/or, consider heavy-atom counts of contacting residues or counts of hydrogen bond and salt bridge opportunities. Decompose by CDR and framework.
4. Claims about specificity from antigen-agnostic predictions are overstated. The manuscript links broader predicted paratopes to higher specificity. Without the antigen, the model identifies where an antibody tends to bind something, not where it binds a given antigen. Suggestion: Pare down the language to association. Add a comparison against an antigen-specific predictor on a matched subset. Report precision and recall trade-offs, average predicted paratope extent, and binder versus non-binder separability.
5. Paratope-weighted embeddings for affinity regression are not consistently beneficial. The regression results show mixed and often small gains across protein language models and datasets. Some models degrade in some datasets. The current text reads stronger than the evidence. Suggested fix: Report confidence intervals and paired tests for changes in R^2 and rank correlations, especially for Table 4. You can consider using subsampling to create confidence intervals. Keep stronger claims for the classification results where significance is shown and soften the regression claims. Discuss the assumptions, such as disregarding the potential allosteric regulation of binding throug residues that are distal to the paratope.
6. Statistical reporting needs to be more explicit in key figures. Some panels, like 4B, show large variance with very small p values and omit test details.

Minor

1. Why was Paragraph not tested with AF2 as well? Is ABB known to be a higher quality predictor than AF2 for antibodies? If so, please cite papers that show this. Otherwise, please report Paragraph results with AF2 as well.
2. Ensemble size versus practical benefit is not fully justified. The six-PLM ensemble does not dominate on all datasets according to the ablation study and adds computational cost. For example, antiBERTy doesn’t seem to help at all based on table S1. Consider having a final model without it (and perhaps also a few others ablated if helpful).
3. The hybrid model routing for Paraplume-G needs more clarification. The description of where each component applies by region and how exactly they’re fused can be supplemented by a schematic.
4. Show absolute value symbols when plotting magnitude changes.
5. Fig 4 has the legend overlapping between 4B and 4E.
6. Putting the codebase on GitHub or an equivalent could make it easier for the reviewers and future users of this work. Zenodo is typically for large datasets associated with the work.

Overall suggestion and conclusion

- The paper is close to publishable with focused revisions on important claims. I recommend a major revision. The central biological claims should be reanalyzed with corrected germline handling or with strong sensitivity analyses. The asymmetry upper bound should be recomputed on exactly or nearly identical arms or with strict distance controls. The paratope size metric should be replaced or grounded with physical measures. Claims about specificity and regression improvements should be tempered and supported with formal uncertainty. None of these changes requires new experiments. They require careful analysis, clearer reporting, and better phrasing. With these updates, the contribution will be stronger and more reliable.
